# Clinical Evaluation of Fiducial Marker Pre-Planning for Virtual Bronchoscopic Navigation Implantation in Lung Tumour Patients Treated With CyberKnife

**DOI:** 10.3389/fonc.2022.860641

**Published:** 2022-06-16

**Authors:** Ki Man Ku, Bing Lam, Vincent W. C. Wu, Kwok Ting Chan, Chloe Y. Y. Chan, H. C. Cheng, Kamy M. Y. Yuen, Jing Cai

**Affiliations:** ^1^ Department of Health Technology and Informatics, The Hong Kong Polytechnic University, Hong Kong, Hong Kong SAR, China; ^2^ Hong Kong Radiation Therapy Company Limited, Hong Kong, Hong Kong SAR, China; ^3^ Respiratory Medicine Centre, Hong Kong Sanatorium and Hospital, Hong Kong, Hong Kong SAR, China; ^4^ Department of Radiotherapy, Hong Kong Sanatorium and Hospital, Hong Kong, Hong Kong SAR, China; ^5^ Hong Kong Medical Physics Consulting Company Limited, Hong Kong, Hong Kong SAR, China; ^6^ Research Institute for Smart Aging, The Hong Kong Polytechnic University, Hong Kong, Hong Kong SAR, China

**Keywords:** CyberKnife, fiducial marker, virtual bronchoscopic navigation, lung cancer, dose area product

## Abstract

**Purpose:**

For the treatment of invisible lung tumours with CyberKnife (CK), fiducial markers (FMs) were implanted as an internal surrogate under virtual bronchoscopic navigation (VBN). This research aims to study the benefits of introducing an additional procedure in assigning the optimal FM positions using a pre-procedure planning system and performing virtual simulation before implantation. The objectives were 1) to reduce the duration of the FM implantation procedure, 2) to reduce the radiation exposure in dose area product (DAP) (dGy*cm^2^) to patients, and 3) to increase the number of FMs implanted around the tumour.

**Methods and Materials:**

This study is retrospective, single-centre, and observational in nature. A total of 32 patients were divided into two groups. In Group 1, 18 patients underwent conventional VBN FM implantation. In Group 2, 14 patients underwent additional pre-procedure planning and simulation. The steps of pre-procedure planning include 1) importing CT images into the treatment planning system (Eclipse, Varian Medical Systems, Inc.) and delineating five to six FMs in their ideal virtual positions and 2) copying the FM configuration into VBN planning software (LungPoint Bronchus Medical, Inc.) for verification and simulation. Finally, the verified FMs were deployed through VBN with the guidance of the LungPoint planning software.

**Results:**

A total of 162 FMs were implanted among 35 lesions in 32 patients aged from 37 to 92 (median = 66; 16 men and 16 women). Results showed that 1) the average FM insertion time was shortened from 41 min (SD = 2.05) to 23 min (SD = 1.25), p = 0.00; 2) the average absorbed dose of patients in DAP was decreased from 67.4 cGy*cm^2^ (SD = 14.48) to 25.3 cGy*cm^2^ (SD = 3.82), p = 0.01 (1-tailed); and 3) the average number of FMs implanted around the tumour was increased from 4.7 (SD = 0.84) to 5.6 (SD = 0.76), p = 0.00 (1-tailed).

**Conclusion:**

Pre-procedure planning reduces the FM implantation duration from 41.1 to 22.9 min, reduces the radiation exposure in DAP from 67.4 to 25.3 dGy*cm^2^, and increases the number of FMs inserted around the tumour from 4.7 to 5.6.

## Introduction

Lung stereotactic body radiation therapy (SBRT) is an advanced radiotherapy technique that delivers high and ablative doses of radiation to lung cancer patients as well as patients with metastatic lung tumours in an oligometastatic state, where the metastases are limited in number and location, with high precision (1–3). CyberKnife (CK) (Accuray, Inc., Sunnyvale, CA, USA) SBRT system uses a seamless integration of periodic X-ray imaging for internal target tracking integrated with Synchrony, which is an optical image guidance system for external respiratory motion tracking (4–6). Previous studies showed excellent local control at 1- and 2-year follow-ups for patients treated with CK SBRT (7–12).

Effective internal target tracking requires the implantation of metallic fiducial markers (FMs), which act as internal surrogates of the tumour’s position and motion, before radiotherapy planning. The FM implantation procedure was performed under monitored anaesthesia care (MAC) in the Endoscopy Department by respiratory medicine specialists using either virtual bronchoscopic navigation (VBN) (13) or electromagnetic navigation bronchoscopy (ENB) (14). The VBN/ENB is designed as a Global Positioning System (GPS) to guide bronchoscopic tools to the predefined tumour location or until the tumour is visible. Efficiently placing an adequate number of FMs around the tumour can be challenging. When there were no strategies or standardized guidelines on VBN/ENB implantation, doctors have to navigate around the tumour through small bronchi and look for feasible locations under X-ray images or fluoroscopies by a C-arm machine. As a result, the overall procedure time for the FM implantation is prolonged, and the patient is exposed to a large amount of radiation from X-ray images or fluoroscopies. Furthermore, it is difficult for doctors to deploy an ideal amount of 5–6 FMs. Additionally, while searching the related literature, we found very limited references focusing on the standardized FM implantation procedure. Many studies investigated the FM implantation methods and their resulting complications, as well as the marker retention and migration rates (15–17). Some studies investigated the co-relationship between FMs and tumours to predict how well the FM configuration represents the tumour motion and to determine the desirable FM configuration (18, 19). However, few studies have described how to implant the FMs into desirable and appropriate positions and determined the FM configuration that best represents the tumour motion. In a more recent study, investigators showed that using an FM placement guidance system may increase the number of FMs being tracked (20).

To cope with the above difficulties, we introduced additional pre-procedure planning before the FM implantation in March 2019. Pre-procedure planning aims to predefine the proper positions and ideal configurations of the FMs. By doing this, we expected that the time of the FM implantation procedure could be reduced, which resulted in shorter MAC time and less radiation exposure to the patient and staff (20). We also expected that more FMs could be placed around the tumour to better represent the tumour motion (18, 19). This study aims to evaluate whether pre-procedure planning of optimal FM positions could improve the overall efficiency of FM implantation and increase the number of FMs implanted around the tumour. In particular, we try to answer the following questions in our investigation: 1) whether the proposed procedure can reduce the duration of the FM implantation procedure, 2) whether the proposed procedure can reduce the radiation exposure to the patient as well as staff during the FM implantation procedure, and 3) whether we can increase the number of FMs implanted around the tumour.

## Methods and Materials

### Types of the Study and Patient Recruitment

This study is retrospective, single-centre, and observational in nature. The study proposal was submitted to and approved by the Research Ethics Committee of the Hong Kong Sanatorium and Hospital Group, and the Human Subjects Ethics Application Review System of the Hong Kong Polytechnic University. Patients with lung tumours and referred for CK treatment with fiducial tracking and those whose FMs will be implanted using VBN were included in this study. Patients who already had FMs in their lungs from previous CK treatment and those whose FMs will be used again for current CK treatment were excluded from this study. A total of 32 patients with lung tumours were referred for FM implantation using VBN before CK treatment had been selected. They were divided into two groups according to the methods of FM implantation. The first group of 18 patients were those who underwent VBN FM implantation and were treated with CK from June 2017 to August 2019. The second group of 14 patients were those who underwent additional pre-procedure planning before the VBN FM implantation from March 2019 to July 2020.

### Pre-Procedure Planning Steps

**Figure 1:**
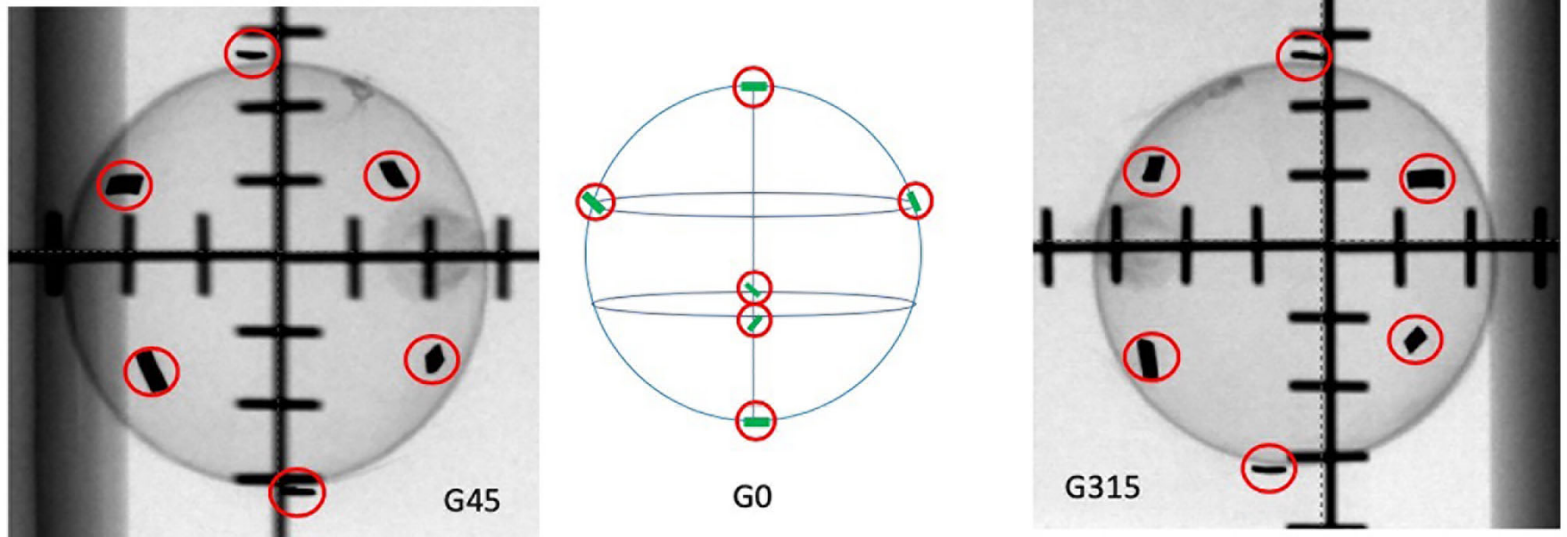
The tested ideal FM configurations. G45 represents the 45° oblique view. G0 is the graphical illustration of how the FMs are ideally distributed on the surface of a sphere. G315 represents the 315° oblique view.

**Figure 2:**
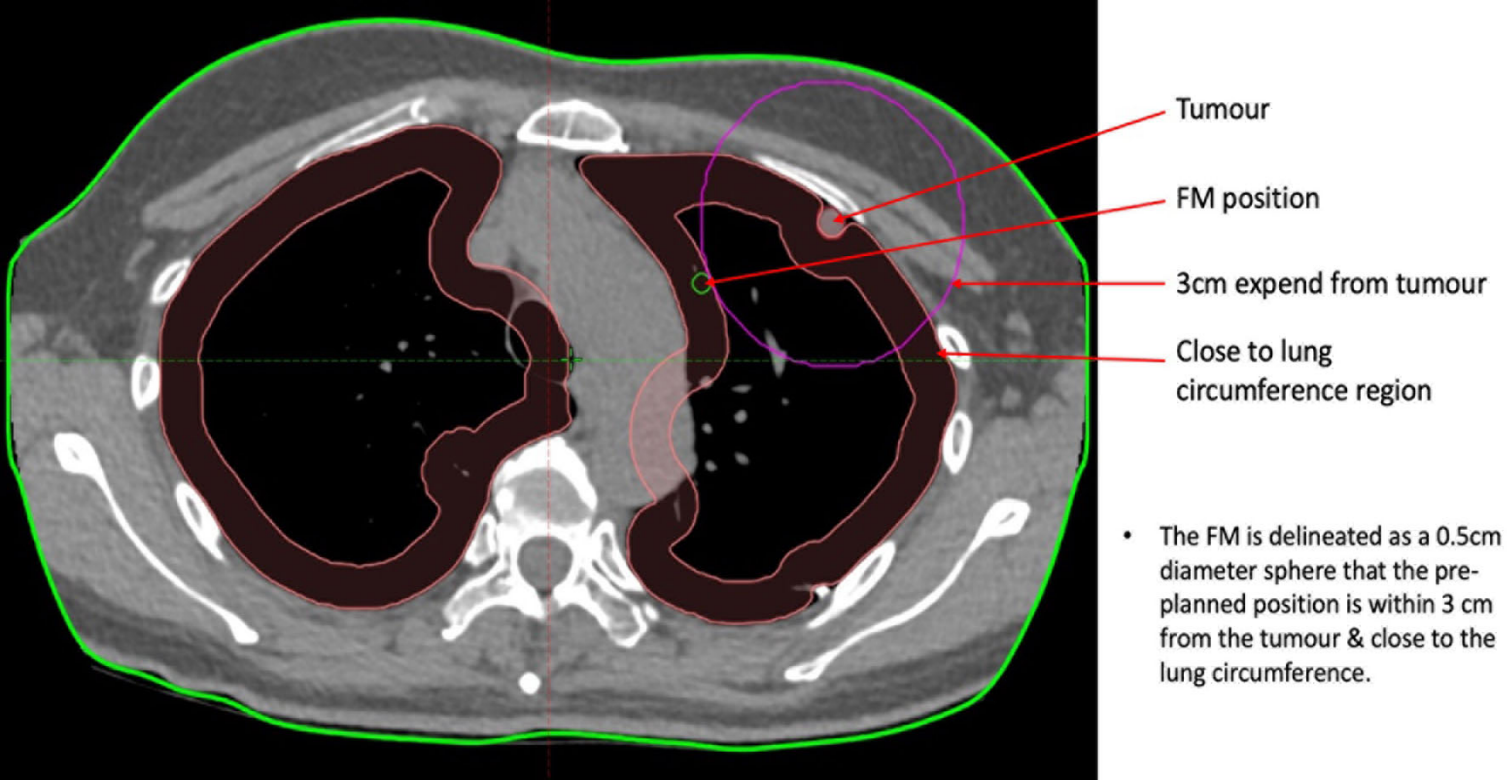
Illustration of the guidelines for the pre-planned FM position.

**Figure 3:**
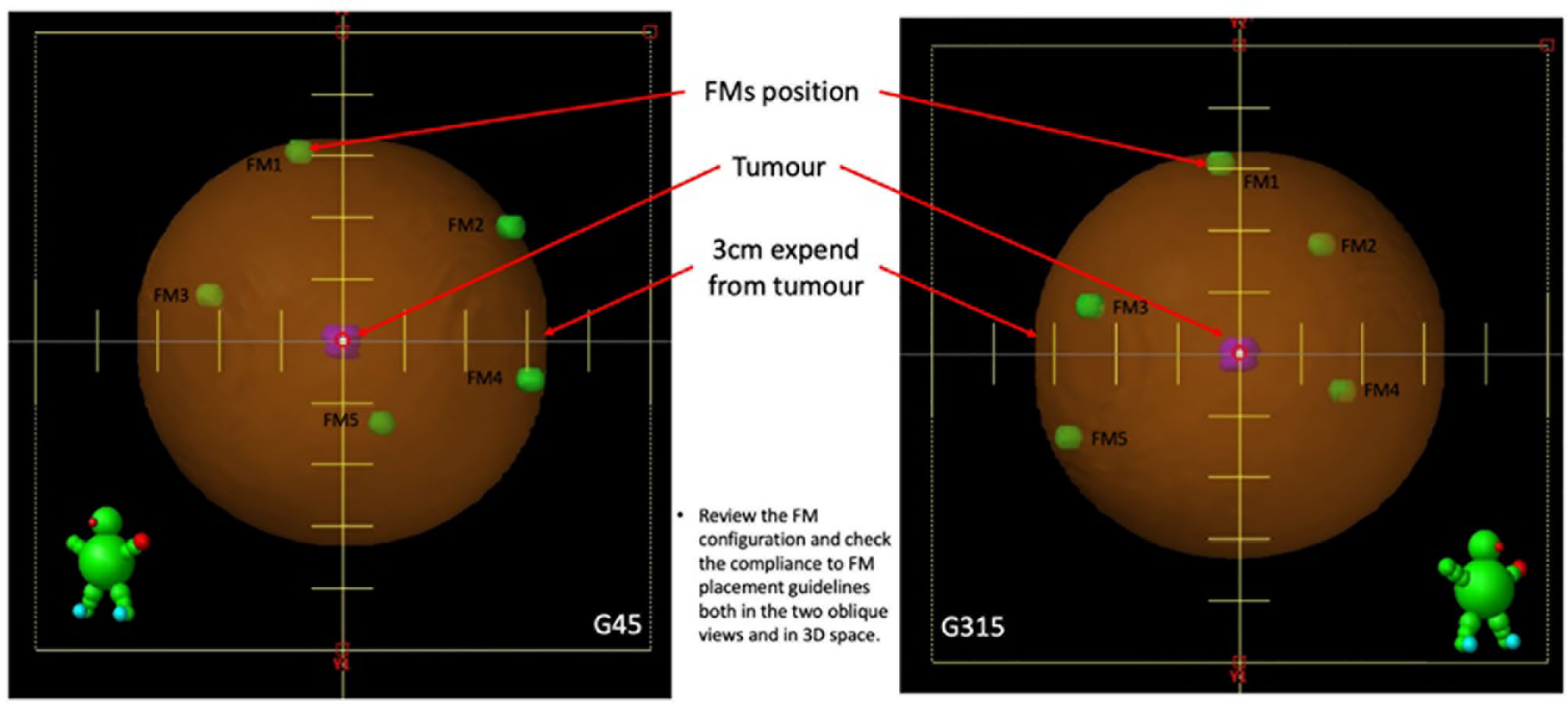
Illustrations of the FM configuration review in the two oblique views.

Pre-procedure planning involves two steps. The first step is to predefine 5–6 optimal FM positions around the tumour on the patient’s CT images in Eclipse (Eclipse, Varian Medical Systems, Inc., Palo Alto, CA, USA) radiotherapy treatment planning system (TPS). The second step is to import the CT image set together with the predefined FM positions into a VBN procedure planning system (LungPoint Bronchus Medical, Inc., San Jose, CA, USA) for verification and simulation. FMs were then deployed through VBN implantation like previously, but this time with the guidance of a procedure planning system. For the second group of 14 patients who underwent pre-procedure planning, a set of low-dose CTs was performed and imported into the Eclipse RT planning system for an oncologist to delineate the preliminary target. After that, 5–6 predefined optimal FM positions were also contoured around the preliminary target. The procedures and guidelines of the FM contouring were as follows:

1. Delineate each of the FMs with a 0.5-cm-diameter sphere around the tumour and then label them as FM1 to FM5/6.

2. Keep the distance between each FM and the tumour between 1 and 3 cm. This can be performed by creating a pseudo-structure by expanding the tumour to 1 to 3 cm, depending on the size of the tumour and contouring the FMs on the circumference of the pseudo-structure. Although the co-relationship between the FMs and the tumour is better if the distance between them is shorter, the distance between any two individual FMs may be too short or less than the 18 mm, which is the minimum inter-FM distance required for CK tracking. Generally, for a tumour volume of 1 cc or less, a pseudo-structure should be created by expanding the tumour by 3 cm. For a tumour volume ranging from 1 to 3 cc, the pseudo-structure should be created by expanding the tumour by 2 cm. For a tumour volume of 3 cc or more, the pseudo-structure should be created by expanding the tumour by 1 cm.

3. Delineate the FMs as distal as possible in the small bronchi to increase the chance of fixation.

4. For tumours located in the middle of the lung with at least 2 cm of circumferential lung tissues, delineate 6 FMs and arrange the FMs, as follows (**Figure 1**):

- Delineate two FMs to the most superior and inferior points of the pseudo-structure created from tumour expansion.
- At a one-third longitudinal position from the superior point, delineate the other two FMs at either the left/right or anterior/posterior points of the pseudo-structure created from tumour expansion.
- Delineate the last two FMs at the two-thirds longitudinal position from the superior point, in a perpendicular position opposing the previous pair and within the pseudo-structure created from tumour expansion.

5. For peripheral lung tumours with at least one side of the tumour not possible for FM implant, delineate 5 FMs and arrange the FMs, as follows (**Figure 2**):

- Delineate two FMs to the most superior and inferior points if the blocked zone is at the circumferential tumour location or to either left/right or anterior/posterior if the blocked zone is located at the cranial–caudal directions, within the pseudo-structure created from the tumour expansion.
- Evenly distribute the remaining 3 FMs at the most distal circumference of the pseudo-structure created from and at the opposite side of the blocked zone of the tumour.

6. Create a set-up plan with two 450° and 3150° oblique fields to simulate the two X-ray imaging views in CK, and review the FM configuration to obtain the following (**Figure 3**):

- The minimum distance between each pair of FMs is larger than 18 mm in three-dimensional (3D) space.
- The minimum angle between a triplet of fiducials should be at least 15°.

7. After the optimal FM positions in the Eclipse RT planning system are defined, import the low-dose CT again into another system, the LungPoint VBN procedure planning system (2018 version, Bronchus Medical, Inc., San Jose, CA, USA).

8. In the LungPoint system, generate the patient’s bronchial tree automatically with the procedure planning system.

9. Copy all the delineated FMs in the Eclipse RT planning system to the LungPoint procedure planning system manually.

10. Review the FMs’ virtual positions to see if they are at or near the end of the most proximal small bronchi (**Figure 4**).

**Figure 4:**
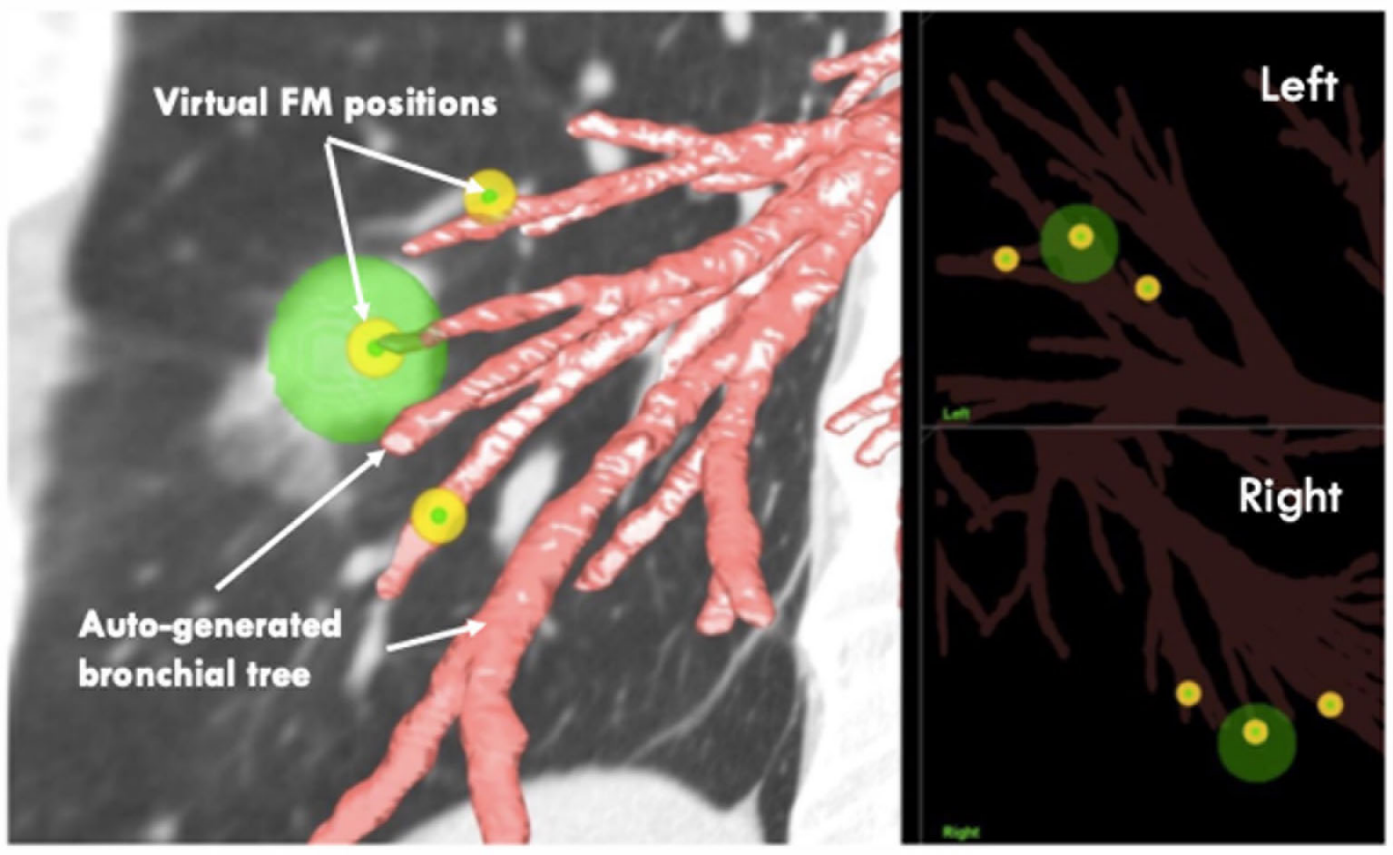
Illustration of the autogenerated bronchial tree and the pre-planned FM positions.

**Figure 5:**
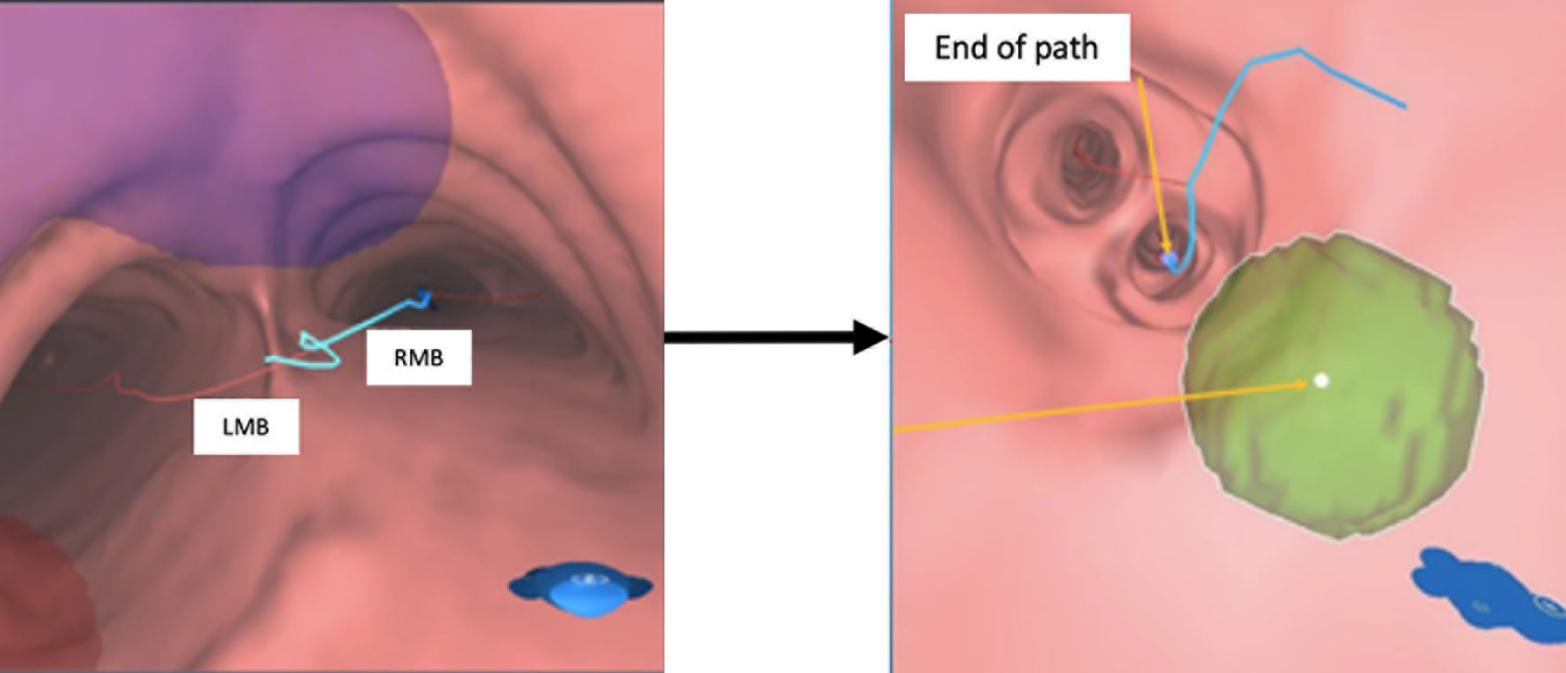
VBN simulation in the pre-planning system. Left: animated simulation of the VBN guidance. Right: at the end of the route, virtual FM positions are reachable. VBN, virtual bronchoscopic navigation.

11. Press the play button. The system will then simulate the VBN process and guide the virtual bronchoscope to the target positions.

12. Assess and confirm that each of the pre-planned FM positions is reachable at the end of the simulation (**Figure 5**).

### Fiducial Marker Implantation

**Figure 6:**
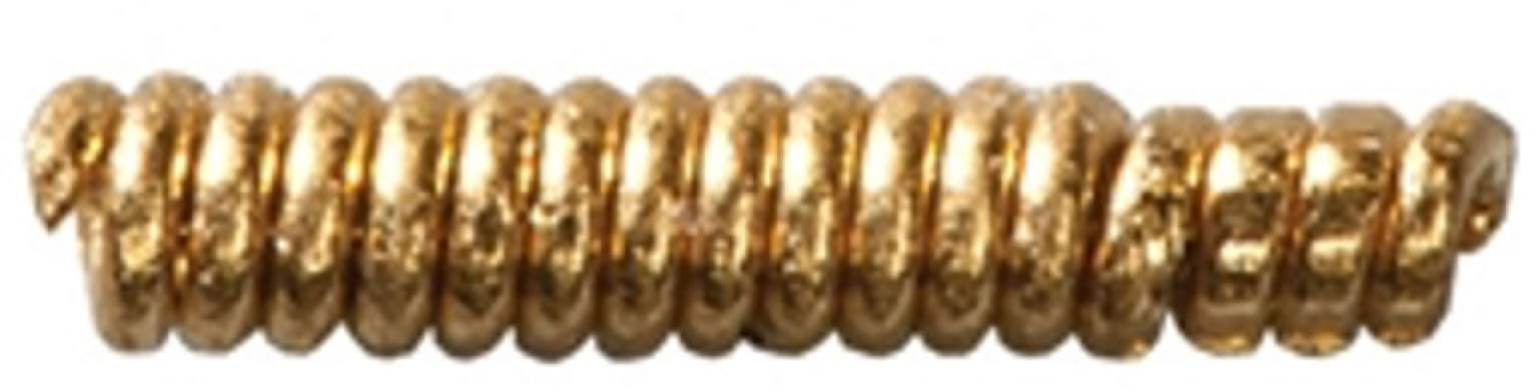
PointCoil™ Marker is a 5-mm-long helical coil for IGRT. The design maximizes stability and minimizes artefacts. IGRT, image-guided radiotherapy.

All FMs, measured 0.8 × 5 mm (PointCoil MTCTXPC08) (**Figure 6**), were then implanted by a respiratory medicine specialist using VBN under the active guidance of the LungPoint procedure planning system.

### Data Collection and Analysis

For the comparison of the FM implantation duration, the total time used for FM implantation with VBN for both groups was recorded. For the comparison of X-ray exposure to patients and staff, the dose area product (DAP) in dGy*cm^2^, a quantity used in assessing the radiation risk from diagnostic X-ray examinations, was collected from the internal chamber of the manufacturer (Axiom Artis, Siemens, Munich, Germany) for each exposure for both groups. Furthermore, the total number of FMs implanted during the VBN session was collected and compared for each patient in both groups.

Data analysis was performed using Data Analyses, an add-in statistical tool of Microsoft Excel (Version 16.50, Microsoft 365 Subscription). A parametric, independent t-test was used to compare the means of the two groups of the patients. All p-values were two-sided. A p-value of less than 0.05 was statistically significant.

## Results

A total of 32 patients (16 men and 16 women aged 37 to 92; median 59.5, SD = 16.58) were recruited for this study, and their demographics are summarized in **Table 1**. Twelve patients’ lesions were centrally located, and 20 patients’ lesions were peripherally located. There were 35 lesions in 32 patients, and a total of 162 FMs were inserted with no procedure-related complications noted.

**Table 1:** Patient demographic information.

Group	Gender	Age	Number of lesions	Side of lung	Location of tumour	Lobe of lung	FMs inserted
1	M	50	1	Rt	Central	RLL	4
1	M	82	1	Rt	Peripheral	RLL	3
1	F	53	1	Rt	Central	RLL	4
1	F	88	1	Rt	Central	RLL	5
1	M	53	1	Lt	Central	LUL	4
1	M	88	1	Lt	Peripheral	LLL	4
1	F	52	1	Lt	Peripheral	LLL	4
1	M	89	1	Rt	Central	RLL	4
1	M	88	1	Lt	Peripheral	LLL	5
1	F	53	1	Rt	Central	RUL	6
1	M	80	1	Rt	Central	RML	5
1	M	91	1	Lt	Peripheral	LUL	5
1	F	52	1	Rt	Peripheral	RML	6
1	M	84	1	Rt	Peripheral	RML	5
1	F	89	1	Lt	Central	LUL	5
1	F	48	1	Rt	Peripheral	RUL	6
1	M	65	1	Rt	Peripheral	RUL	4
1	M	65	1	Lt	Peripheral	LLL	5
2	F	54	2	Rt	Peripheral	RML	5
2	F	54	1	Lt	Peripheral	LLL	4
2	F	54	1	Lt	Peripheral	LLL	4
2	M	92	1	Rt	Peripheral	RLL	5
2	F	44	1	Rt	Peripheral	RUL	6
2	M	68	1	Rt	Peripheral	RLL	6
2	F	59	1	Lt	Peripheral	LLL	6
2	F	59	1	Lt	Peripheral	LUL	6
2	M	77	1	Lt	Central	LUL	6
2	M	52	2	Rt	Central	RUL	6
2	F	60	1	Lt	Peripheral	LUL	6
2	F	37	1	Lt	Peripheral	LLL	6
2	F	73	1	Rt	Central	RLL	6
2	M	52	2	Rt	Central	RUL	6

FMs, fiducial markers; RLL, right lower lobe; LUL, left upper lobe; LLL, left lower lobe; RUL, right upper lobe; RML, right middle lobe.

For the insertion time comparison, the mean, minimum, maximum, and SD values of the procedure duration for both groups of patients are presented in **Table 2**. Results showed that the mean duration of FM implantation was reduced from 41.1 min (minimum 26.0 to maximum 56.0 min) for those without pre-procedure planning (Group 1) to 22.9 min (minimum 15.0 to maximum 30.0 min) for those with pre-procedure planning followed by VBN (Group 2). This difference was significant, p = 0.00 (1-tailed).

**Table 2:** The procedure durations of both groups of patients (min).

	Group 1	Group 2
Mean	41.1	22.9
SD	8.7	4.7
Minimum	26.0	15.0
Maximum	56.0	30.0
Count	18.0	14.0

**Figure 7:**
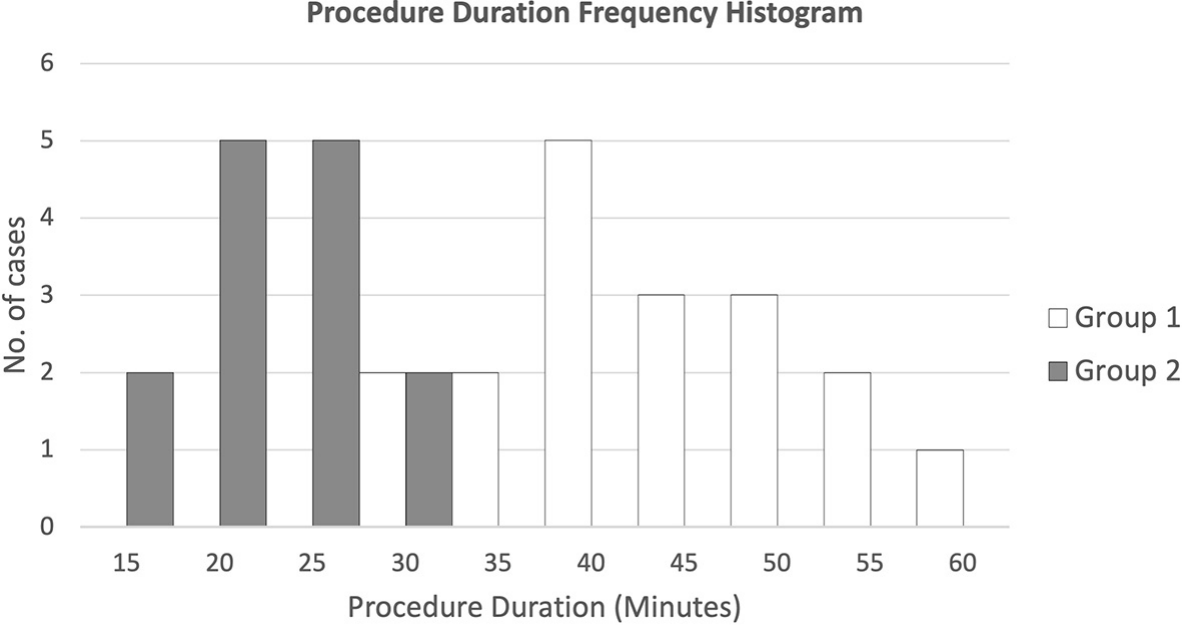
Procedure duration frequency histogram for both groups of patients.

**Figure 7** presents the duration frequencies of FM insertion for both groups of patients through a histogram. Results showed that for FM implantation without pre-procedure planning, most of the durations were from 35 to 55 min, while for FM implantation with additional pre-procedure planning, all durations ranged from 15 to 30 min.

For the radiation exposure comparison, the mean, minimum, maximum, and SD values of the DAP (dGy*cm^2^) are presented in **Table 3**. Results showed that the mean DAP of FM implantation was reduced from 67.4 (minimum 10.9 to maximum 217.6 dGy*cm^2^) for those without pre-procedure planning (Group 1) to 25.3 (minimum 9.0 to maximum 47.2 dGy*cm^2^) for those with pre-procedure planning followed by VBN (Group 2). This difference was significant, p = 0.01 (1-tailed).

**Table 3:** The DAP of both groups of patients (dGy*cm^2^).

DAP (dGy*cm^2^)	Group 1	Group 2
Mean	67.4	25.3
SD	56.1	13.8
Minimum	10.9	9.0
Maximum	217.6	47.2
Count	18.0	14.0

DAP, dose area product.

**Figure 8:**
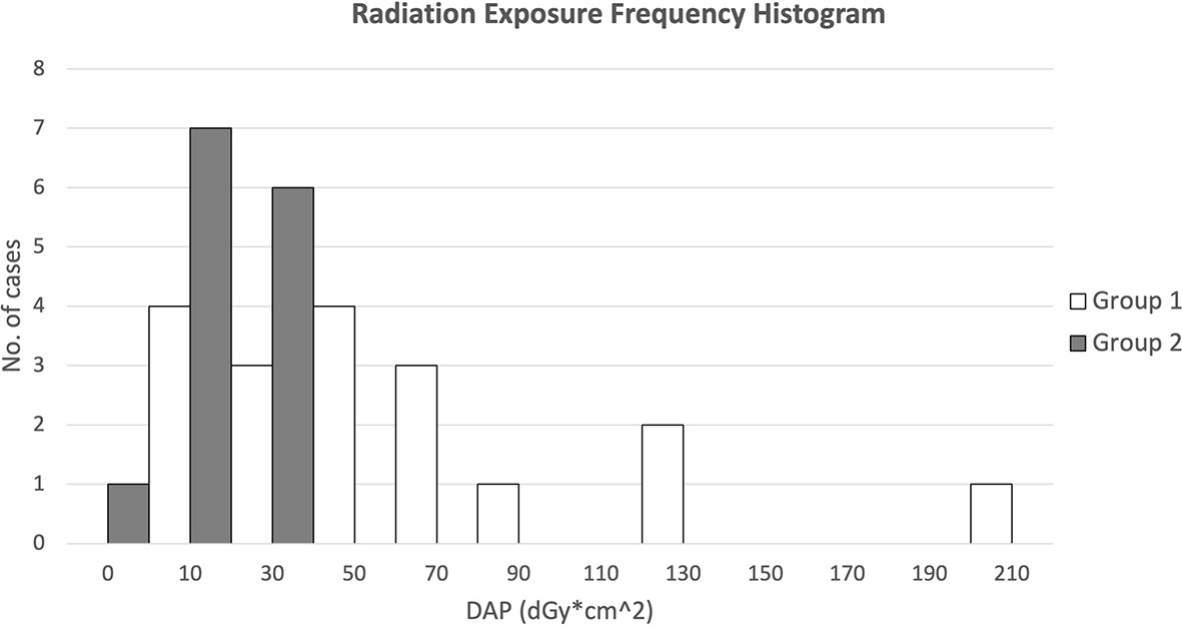
The radiation exposure frequency histogram of both groups of patients (dGy*cm^2^).

**Figure 8** presents the DAP frequency of FM insertion for both groups of patients through a histogram. Results showed that for FM implantation without pre-procedure planning, the DAPs were widely spread from 10 to 130 with 1 extreme case of more than 200 dGy*cm^2^, while for FM implantation with additional pre-procedure planning, all DAPs were confined within 10 to 50 except one with 9.0 dGy*cm^2^.

For the number of FMs implanted, the mean number of FMs inserted was increased from 4.7 for implantation without pre-procedure planning to 5.6 for implantation with additional pre-procedure planning. Meanwhile, the percentage of patients inserted with 5 to 6 ideal numbers of FMs was increased from 56% to 86%. This difference was significant, p = 0.00 (1-tailed).

**Figure 9:**
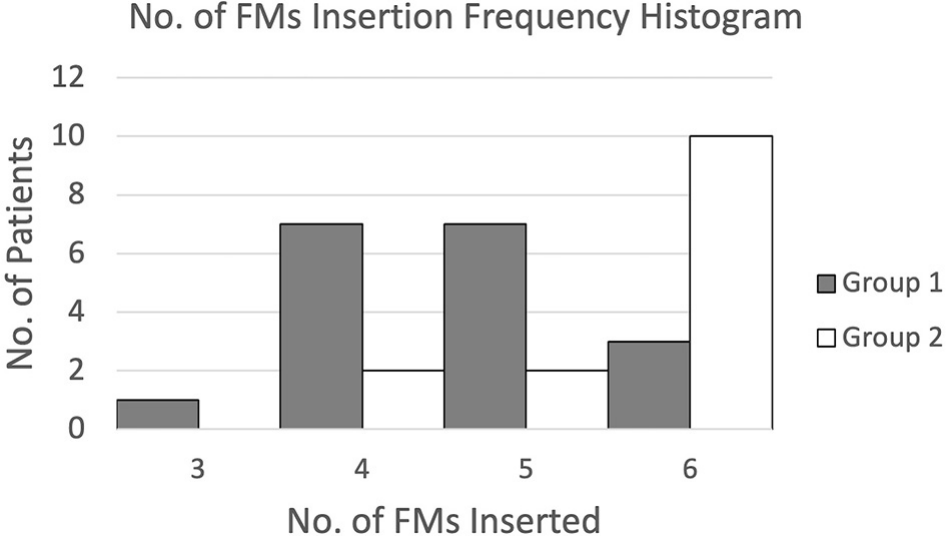
Histogram of the number of FM insertion frequency.

**Figure 9** presents the frequencies of FMs inserted for both groups of patients through a histogram. Results showed that for FM implantation without pre-procedure planning, the number of FMs inserted was widely spread from 3 to 6 and mostly 4 or 5, while for FM implantation with additional pre-procedure planning, most patients were inserted with the maximum amount of 6 FMs.

## Discussion

Respiratory motion is a major challenge for precision radiotherapy of lung cancer (21). Studies have shown that inaccurate tumour motion measurement and tracking can lead to errors in target volume determination and subsequently dose delivery (22, 23). FMs allow for reliable and accurate determination of the lung tumour’s position and motion, and they have the potential to significantly improve treatment safety and outcome (24). In our centre, FM implantation is an essential procedure for all patients with invisible lung lesions and to be treated with CK. In this study, we aim to use an additional procedure before FM implantation to improve the overall efficiency by reducing the time for implantation and radiation exposure to the patient and staff, as well as the quality of the implantation by increasing the number of FMs deployed around the lesion, which means a higher chance of trackability more representative of the tumour motion (25).

### Tolerability and Complication

FMs can be implanted into the lung using two methods, transthoracic/percutaneous and transbronchial through the patient’s respiratory tract. Research showed that percutaneous or CT-guided transthoracic insertion of FMs is associated with a high risk of pneumothorax (16, 26–28). In contrast, transbronchial FM implantation is much safer. A review of 5 studies using the transbronchial FM implantation method showed that 4 of them recorded 0% implantation-induced pneumothorax (14, 15, 29, 30), and the remaining one recorded 2.3% (31). Other recent studies showed that the rate of complication is usually associated with guided transbronchial biopsies (TBBs) performed before FM implantation (32, 33). In this study, there was neither bleeding nor pneumothorax in both groups of our patients after FM implantation with or without pre-procedure planning.

### Improving the Fiducial Marker Insertion Efficiency

Previous FM insertions were complicated due to the absence of pre-procedure planning. The procedure is dependent on the experience and real-time decision making of a group of different health professionals, including an anaesthetist handling the patients’ condition, a respiratory specialist manipulating the bronchoscope and navigating inside patients’ bodies to look for a good position for the FM, a medical physicist, and a radiation therapist from the CK team staying in the control room, giving advice and confirming the location for FM deployment. With pre-procedure planning, we can decide where and how many FMs can be inserted and what will be the overall resulting configuration. We will also be aware of the possibility that one or more directions could not be feasible for FM deployment or that there are no small bronchi for FMs to be firmly anchored to. Thus, the FM insertion procedure becomes simpler.

The results of this study show that with pre-procedure planning, the average time of FM insertion can be reduced from 41.1 to 22.9 min or by 44% (median = 22.5 min). Furthermore, the overall procedure duration is more predictable, where 9 out of 14 patients’ procedure times are within 20 to 25 min and the SD is reduced from 8.7 to 4.7 min. Improving efficiency brings several advantages. First, the time of patients under anaesthesia can be reduced. It is expected that the risk due to anaesthesia will also be reduced, and hopefully, this procedure can be tolerable by more patients. Second, utilization of the endoscopy room can be increased. With a shorter and more predictable duration, resource allocation will be more effective, and more patients can be arranged for endoscopy procedures. Furthermore, the cost of the procedure is expected to be reduced. The endoscopy room is one of the costliest components of a hospital, and a reduction in the procedure time is expected to be followed by a reduction in cost to patients, which results in a higher chance of continuing to enjoy organizational success.

In a more recently published study by Casutt et al. (2021) (33), the researchers evaluated FM implantation procedures using endobronchial insertion under fluoroscopy like ours but without pre-procedure planning. Their results showed that the median time of the procedure was 31.5 min (10-95 min). Our study showed a shorter duration (22.5 vs. 31 min) and a more consistent range of time (15–30 vs. 10–90 min). In addition, the average number of FMs implanted in Casutt’s study was 3.0, while in our study, it was 5.6 with pre-procedure planning. The comparison reinforced that pre-procedure planning can improve FM implantation efficiency.

### Reducing the Radiation Exposure

The main principle of radiation protection is to protect patients from unnecessary radiation and perform medical procedures with as low as reasonably achievable (ALARA) doses. In this study, we use the DAP measured during the examination as a quantitative tool to compare the radiation exposure of patients as well as staff during the FM insertion procedure.

The results of this study show that with pre-procedure planning, the average DAP is reduced from 67.4 to 25.3 dGy*cm^2^ or by 62%. Furthermore, the overall radiation exposure to patient and staff become more consistent and predictable, which is shown by the reduced SD from 56.1 dGy*cm^2^ in Group 1 patients to 13.8 dGy*cm^2^ in Group 2 patients. This is largely due to the reduction in time of fluoroscopy. Because the location of FMs is predefined and the route to the designated location is guided by the VBN system, respiratory specialists can avoid frequent fluoroscopy during the procedure to locate the position of the endoscope and look for ways to the desired location. Furthermore, staff radiation exposure is expected to be reduced too, which also reduces the chance of staff overexposure resulting in manpower shortage.

### Increasing the Number of Fiducial Markers Implanted

The lung tumour moves in all directions, and the amplitude can be up to 12 mm in the cranial–caudal direction (16). According to the CK guideline on FM placement, at least 3 FMs are required for 3D tumour tracking, while 4–6 are recommended for more secure and confident tumour motion tracking along with the CK treatment (34). Meanwhile, researchers also recommended implanting 4–6 FMs to improve marker reliability and tracking accuracy (35).

The results of this study show that the average number of FMs inserted only increased slightly from 4.7 to 5.6 with the application of pre-procedure planning. However, with the new workflow, 10 out of 14 patients can be inserted with 6 FMs, and no patient will be inserted with less than 4 FMs. This largely increases the reliability of the FMs in representing the true tumour motion and the confidence of continuous 3D tracking along with the treatment in considering the possibilities of FM migration.

### The Effect on Target Trackability

Theoretically, we could expect better trackability based on the concept that with pre-procedure planning, the ideal locations of the FMs were preliminarily identified. The ideal locations could fulfil both the criteria that it should be at the distal end of small bronchi and located at a predefined distance away from the tumour, making a good overall configuration around the tumour, which means a higher chance of trackability. In reviewing this, we defined the trackability of the target or FMs by the number of FMs implanted to the number of FMs tracked when the CK treatment starts and compare the results of the two groups of patients.

Results showed that the trackability was increased from 41% (minimum 0% to maximum 75%) for patients without pre-procedure planning (Group 1) to 50% (minimum 33% to maximum 100%) for patients with pre-procedure planning (Group 2). However, the difference was insignificant, p = 0.12 (1-tailed). This could be explained by the patient-dependent nature of the probability of FM migration. For patients suffering from frequent coughing due to lung disease, migration or even loss of FMs is common.

### The Values of This Study

FM tracking is the only choice for CK of non-small cell lung cancer (NSCLC) patients with small tumours that are invisible under the planar X-ray view. Real-time fiducial tracking together with Synchrony respiratory motion management results in excellent motion synchronized treatment with sub-millimetre margins to the targets (17). This study introduced a new set of implantation rules and guidelines that could become a helpful reference for other clinical centres using CK in SBRT. We expect that if the suggested pre-planned FM positions method is proved to be more effective, FM implantation procedures will be well-organized, and the results will be more predictable.

### Assumptions and Limitations of the Study

There are some assumptions for this study. First, it is not possible to move all FMs in the same vectors and not cause differences in the FM centroid and displacement. Second, tumour shrinkage during the period of CK treatment is negligible. Although one previous study showed that the tumour will shrink during SBRT, the shrinkage rates were not necessarily uniform (18). In the present study, it is assumed that the size of the tumour remains unchanged. Therefore, all the FM displacements that will be measured after the CT images are fused are due to FM displacement.

**Figure 10:**
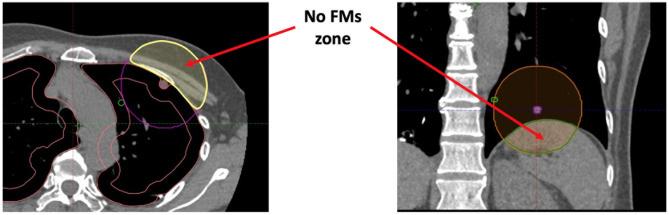
The illustration of no-FM zone if the lung tumour is close to the lung circumference.

This study also has some limitations. First, although an ideal FM configuration can be determined, it is nearly impossible to achieve. If the lung tumour is located near the lung circumference of the diaphragm, there will be a no-FM zone such as the chest wall and the diaphragm, which limits the all-around distribution of the FMs, resulting in an unevenly distributed FM arrangement and increasing the distance between the tumour and the FM centroid (**Figure 10**). Second, it is not well-known how close the VBN combined with ultrathin endoscopy-guided FM implantation can be to the pre-planned FM positions. It is assumed that the discrepancies between the two can be within 1 cm. Third, only small bronchi near the lung circumference are highly FM fixed. Therefore, only about one-third of the lung volumes are possible for FM implantation. Moreover, to some extent, the fixation of FMs was patient dependent. For example, coughing is one cause of FM migration. Patients with a coughing problem during the period of CK treatment may have a higher chance of FM migration and displacement. Finally, some patients have more than one tumour located close to each other. A set of FMs can be used to treat a combination of one or more tumours at the same time, but this will complicate the definition of the tumour centroid and the distance of the FM to the tumour.

### Future Directions

The CK FM tracking system is complex. To achieve 6DOF FM tracking, criteria such as minimum distance between FM angles between FM configuration should all be fulfilled. However, there were no technical guidelines on how to implant FMs in fulfilling the CK tracking criteria. Furthermore, FMs cannot easily be fixed in the small bronchi after implantation. Future studies should focus on increasing FM stability and fixation by using different kinds of FMs and how to implant FMs into a designated position accurately. Moreover, future similar studies should consider increasing their sample size.

## Conclusion

In this study, implementing additional pre-procedure planning before FM implantation improves the overall efficiency by reducing the duration from 41.1 to 22.9 min. Meanwhile, the new workflow reduces the radiation exposure by decreasing the DAP from 67.4 to 25.3 dGy*cm^2^. Furthermore, the number of FMs inserted around the tumours is increased from 4.7 to 5.6, and the number of patients inserted with 5 or 6 FMs is increased from 56% to 86%.

## Data Availability Statement

The raw data supporting the conclusions of this article will be made available by the authors, without undue reservation.

## Ethics Statement

The studies involving human participants were reviewed and approved by Hong Kong Sanatorium and Hospital Medical Group Research Ethics Committee, and Hong Kong Polytechnic University Ethics Committee. Written informed consent for participation was not required for this study in accordance with the national legislation and the institutional requirements.

## Author Contributions

KK proposed and developed the idea and wrote the manuscript. BL performed the procedure and helped obtain ethical approval. VW reviewed and commented on the proposal. KC and YC were responsible for data collection. HC and KY were responsible for data analysis. JC revised the manuscript and played a major role as a supervisor. All authors listed have made a substantial, direct, and intellectual contribution to the work and approved it for publication.

## Conflict of Interest

Authors KK and KY were employed by Hong Kong Radiation Therapy Company Limited. Author HC was employed by Hong Kong Medical Physics Consulting Company Limited.

The remaining authors declare that the research was conducted in the absence of any commercial or financial relationships that could be construed as a potential conflict of interest.

## Publisher’s Note

All claims expressed in this article are solely those of the authors and do not necessarily represent those of their affiliated organizations, or those of the publisher, the editors and the reviewers. Any product that may be evaluated in this article, or claim that may be made by its manufacturer, is not guaranteed or endorsed by the publisher.
